# C9ORF72 poly-PR disrupts expression of ALS/FTD-implicated *STMN2* through SRSF7

**DOI:** 10.1186/s40478-025-01977-2

**Published:** 2025-03-26

**Authors:** Karen S. Wang, Julie Smeyers, Kevin Eggan, Bogdan Budnik, Daniel A. Mordes

**Affiliations:** 1 Institute for Neurodegenerative Diseases, University of California, San Francisco, CA USA; 2 Department of Pathology, University of California, San Francisco, CA USA; 3 Mass Spectrometry and Proteomic Laboratory, FAS Division of Science, Harvard University, Cambridge, MA USA; 4 Department of Stem Cell and Regenerative Biology, Harvard University, Cambridge, MA USA; 5 Stanley Center for Psychiatric Research, Broad Institute of MIT and Harvard, Cambridge, MA USA; 6 Department of Pathology, Massachusetts General Hospital, Boston, MA USA; 7 Wyss Institute, Harvard University, Boston, MA USA

## Abstract

A hexanucleotide repeat expansion in C9ORF72 is the most common genetic cause of amyotrophic lateral sclerosis (ALS), frontotemporal dementia (FTD), and combined ALS/FTD. The repeat is transcribed in the sense and the antisense directions to produce several dipeptide repeat proteins (DPRs) that have toxic gain-of-function effects; however, the mechanisms by which DPRs lead to neural dysfunction remain unresolved. Here, we observed that poly-proline-arginine (poly-PR) was sufficient to inhibit axonal regeneration of human induced pluripotent stem cell (iPSC)-derived neurons. Global phospho-proteomics revealed that poly-PR selectively perturbs nuclear RNA binding proteins (RBPs). In neurons, we found that depletion of one of these RBPs, SRSF7 (serine/arginine-rich splicing factor 7), resulted in decreased abundance of STMN2 (stathmin-2), though not TDP-43. STMN2 supports axon maintenance and repair and has been recently implicated in the pathogenesis of ALS/FTD. We observed that depletion of SRSF7 impaired axonal regeneration, a phenotype that could be rescued by exogenous STMN2. We propose that antisense repeat-encoded poly-PR perturbs RBPs, particularly SRSF7, resulting in reduced STMN2 and axonal repair defects in neurons. Hence, we provide a potential link between DPRs gain-of-function effects and STMN2 loss-of-function phenotypes in neurodegeneration.

**Supplementary Information:**

The online version contains supplementary material available at 10.1186/s40478-025-01977-2.

## Introduction

ALS is a rapidly progressive neuromuscular disease that shares clinical and pathological features with another fatal disease, frontotemporal dementia (FTD) [1]. The most common genetic cause of ALS and FTD is a hexanucleotide (GGGGCC) repeat expansion in a noncoding region of *C9orf72* [2–4]. The repeat region is transcribed and non-canonically translated both in the sense and antisense directions into distinct dipeptide repeat proteins (DPRs), such as poly-proline-arginine (poly-PR) [5–7]. In model systems, expression of certain DPRs result in detrimental gain-of-function effects on diverse cellular pathways [8–12]. For instance, arginine-rich DPRs perturb membrane-less organelles, including within the nucleus, and poly-PR can induce nucleolar stress [13, 14]. Although the accumulation of DPRs is thought to be a major driver of disease pathogenesis, how DPRs perturb RNA processing in ALS and FTD and contribute to the gradual loss of neurons remains unresolved [8].

Recent clinicopathological studies have provided insight into the temporal progression of C9-ALS/FTD, suggesting that neurodegenerative processes may begin and unfold over a decade prior to the onset of symptoms [15]. Presymptomatic C9ORF72 repeat expansion carriers exhibit neuroimaging evidence of cortical atrophy and elevation of the plasma biomarker neurofilament light chain (NfL), occurring possibly as early as 30 years prior to disease onset [15]. In a rare circumstance of serial brain sampling in a repeat expansion carrier, a cortical biopsy was taken years prior to the onset of FTD symptoms and demonstrated abundant DPR pathology in the absence of TDP-43 inclusions, which were later detected upon autopsy [16]. Several reports have demonstrated that C9-ALS/FTD patient brains may occasionally have widespread DPR inclusions with no detectable or minimal TDP-43 pathology despite extensive neuropathological examination [16–19]. Overall, this suggests that DPRs could be sufficient to induce neural dysfunction that precedes or possibly acts in parallel with TDP-43 dysfunction.

TDP-43, an RNA-binding protein, regulates the processing of hundreds of transcripts and one of its key targets is *STMN2*, an abundantly expressed neuronal-enriched transcript that encodes for a critical regulator of microtubule stability [20, 21]. In rodent models, *Stmn2* is dramatically upregulated in regenerating axons following injury [22]. In neuroblastoma cells and human pluripotent stem cell-derived motor neurons, siRNA-mediated knockdown of TDP-43 leads to decreased *STMN2* expression that is sufficient to impair axon re-growth after axotomy [23, 24]. More specifically, loss of TDP-43 function induces aberrant splicing of *STMN2* through the inclusion of a cryptic exon (CE), resulting in premature poly-adenylation and a truncated transcript [23–25]. Furthermore, recent exciting mouse studies demonstrate that partial or complete loss of Stmn2 is sufficient to impair axonal structure and cause age-dependent deficits in motor function, notably in the absence of any TDP-43 pathology [26–28]. Hence, preservation of *STMN2* expression is important for neuron function and resiliency to injury.

Reduced *STMN2* expression is found in sporadic ALS and C9ORF72-ALS/FTD and potentially in additional neurodegenerative diseases [23]. Although the detection of truncated CE *STMN2* has been generally correlated with the burden of TDP-43 pathology [29], one recent study observed that the expression of full-length *STMN2* did not correlate with levels of phosphorylated TDP-43 [30]. Additionally, a reduction in *STMN2* has been found in brains in Parkinson’s disease, which is not a TDP-43 proteinopathy [31]. Though there are several possible explanations, these observations suggest that additional RNA processing factors may govern the expression of *STMN2* [30]. However, whether there are additional regulators of *STMN2* expression in neurons has not been examined.

Here, we observed that poly-PR results in decreased expression of *STMN2* in human excitatory neurons. Through unbiased global phospho-proteomics, we identified several RNA binding proteins that are perturbed by poly-PR. We found that depletion of one of these RNA processing factors, SRSF7, is sufficient to decrease STMN2 and reduce neural regenerative capacity. Hence, we provide a point of potential convergence between C9ORF72-ALS/FTD DPRs and loss of STMN2 function.

## Materials and methods

### Cell culture

#### iPSCs

The usage of human induced pluripotent stem cells (iPSCs) was approved by the Human Gamete, Embryo and Stem Cell Research (GESCR) Committee at UCSF.

The NGN2-inducible piggyBac-containing iPS3 cell line (hDFn 83/22 iNgn2#9 [iPS3]) was originally described in Nehme et al. The NGN2-inducible piggyBac-containing iPSC line KOLF2.1 J was obtained from Jackson Laboratory. Stem cells were cultured in StemFlex Medium (Life Technologies, A3349401) on Geltrex-coated (LDEV-Free, hESC-Qualified Geltrex basement membrane matrix, Gibco, A1413301) tissue cell culture plates at 37ºC in 5% CO_2_. ROCK inhibitor (Y-27632, custom synthesis) was added to media at the time of passaging and removed the next day.

#### NGN2 neuron differentiation

Patterned induced neurons were differentiated through a combination of doxycycline to rapidly induce NGN2 expression and small molecule patterning factors as previously described (Pintacuda et al.) [32], with the exceptions of excluding normocin or geneticin selection. On day 5–7 post-induction, neurons were passaged via accutase treatment onto plates coated with PLO/laminin (poly-L-ornithine hydrobromide, Sigma, P3655; laminin, Life technologies, 23,017,015) in neuron maintenance medium [neurobasal media (Gibco), 0.2% N-2 supplement (Life technologies), 2% B-27 supplement (Life technologies), 1% Glutamax, 1% MEM non-essential amino acids (NEAA) solution (Gibco)] supplemented with 1:10,000 doxycycline, neurotrophic factors: 1:10,000 brain-derived neurotrophic factor (BDNF), 1:10,000 ciliary neurotrophic factor (CNTF), 1:10,000 glial cell-derived neurotrophic factor (GDNF) (Bio-techne) and, to remove any residual proliferating cells, EdU (5-ethynyl-2’-deoxyuridine) or FUdR (5-Fluoro-2′-deoxyuridine) at 2 uM was briefly added to cultures. On day 7 and onwards, neurons were fed by replacing half of the media with neuron maintenance medium supplemented with neurotrophic factors, every 2–3 days.

#### Cell lines

HEK 293 T cells were cultured in DMEM with 10% FBS (Takara, 631,106 or Fisher Cytiva HyClone, SH3008803). For the transfection of HEK cells, plasmids expressing DPRs fused with GFP or GFP alone, as previously described (Lee et al.) [14], were diluted in Opti-MEM media and transfected with polyethylenimine (PEI, Sigma, 408,727) in 10-cm plates for proteomic experiments or Lipofectamine 3000 reagent (Invitrogen) according to the manufacturer’s standard protocol for additional experiments. Cells were washed in PBS and then briefly trypsinized and immediately pelleted and flash frozen for storage at -80C for subsequent proteomics, or directly lysed, three days post transfection.

All cell lines were routinely tested for mycoplasma with the MycoAlert PLUS Mycoplasma Detection Kit (Lonza, LT07-703), and were negative.

### Phospho-proteomics

The phospho-proteomics and bulk proteomics was performed at Harvard Center for Mass Spectrometry (HCMS). S-trap mini columns were used (Protifi, NY) according to manufacturer protocol. Briefly, samples were resolubilized in 5% SDS for reduction and alkylation, further digested in buffer trypsin (Promega) for 5 h. The digested samples were enriched by High-Select™ TiO2 Phosphopeptide Enrichment Kit (Thermo-Fisher) according to the vendor’s instructions. Enriched phosphopeptides were labeled with TMT16plexPRO (Thermo-Fisher) according to manufacturer protocol. Sample fraction was submitted for single LC–MS/MS experiment that was performed on a Q Exactive HF (Thermo, MA) equipped with 3000 Ultima Dual nanoHPLC pump (Thermo). The Q Exactive HF was operated in data-dependent mode for the mass spectrometry methods. The mass spectrometry survey scan was performed in the Q Exactive HF Orbitrap in the range of 450 –950 m/z at a resolution of 6 × 104, followed by the selection of the twenty most intense ions TOP10 ions were subjected to HCD MS2 event in Orbitrap. The fragmentation isolation width was 0.8 m/z, AGC was 50,000, the maximum ion time was 150 ms, normalized collision energy was 32 V and an activation time of 1 ms for each HCD MS2 scan was set. For phospho-peptides with multiple PTMs, phospho-residues may not necessarily be unambiguously assigned.

### Bulk proteomics

Following the trypsin digest, samples were labeled with TMT6plex (Thermo-Scientific, MA) according to manufacturer protocol. Then, all labeled samples been combined into single sample, and then further desalted and separated by HipH cartridges (Thermo-Scientific, MA) into 20 fractions. Fractions were dried in speedvac (Eppendorf, Germany) and resolubilized on 0.1% formic acid in water buffer for injections into mass spectrometer for analysis of all fractions, which were submitted for single LC–MS/MS experiment as described above for phospho-proteomics.

### Mass spectrometry analysis

Raw data were analyzed in Proteome Discoverer 3.0 (Thermo Scientific) software with Byonic 3.5 node and ptmRS node. Assignment of MS/MS spectra was performed using the Sequest HT algorithm by searching the data against a protein sequence database including all entries from the Human Uniprot database. A MS2 spectra assignment false discovery rate (FDR) of 1% on protein level was achieved by applying the target-decoy database search. Filtering was performed using a Percolator (64bit version) (Kall et al., 2008). For quantification, a 0.02 m/z window centered on the theoretical m/z value of each the six reporter ions and the intensity of the signal closest to the theoretical m/z value was recorded. Reporter ion intensities were exported in result file of Proteome Discoverer 2.4 search engine as an excel tables. The exact place of phosphor moiety was analyzed by ptmRS program (Taus et al., 2011). Differentially expressed proteins between sample groups were analyzed using R script programs based on Bioconductor (https://www.bioconductor.org/). Statistical analysis for differentially expressed proteins was based on peptide level to find changes of phosphor moiety presence in peptides that are statistically significant between two sets. For bulk proteomics, Proteome Discoverer searches were performed with set modification of TMT labeling to each N-terminus of the peptide and side chain of lysine as well as carbamidomethyl modification to cysteine amino acids, and variable modification to allow methionine oxidation and protein N-terminus acetylation. All searches were filtered to 0.1% FDR at protein and peptide levels. Gene ontology enrichment analysis was performed on the g:Profiler toolset server.

### Modulation of gene expression

Pooled siRNAs (SMARTpool) targeting transcripts encoding RNA binding proteins, STMN2, and non-targeting control siRNAs (ON-TARGETplus) and individual siRNAs (ON-TARGETplus modified to minimize off-target effects) were obtained from Horizon Discovery-Dharmacon. Lyophilized siRNAs were resuspended in diluted 5X siRNA Buffer (Horizon Discovery-Dharmacon, B-002000-UB-100) at 20 uM, aliquoted, and stored at -80C. For knockdown experiments, induced neurons were transfected using Lipofectamine RNAiMAX (Invitrogen) with pooled siRNAs (SMARTpool) as a single dose and then harvested for RNA 72 h later, or with individual siRNAs in two doses (0, 48 h) and then harvested at 96 h later. The most effective individual siRNA targeting SRSF7 was compared to a non-targeting control siRNA for functional studies. Total RNA was prepared from cells or neurons using the RNeasy Plus Mini Kit (Qiagen, 74,134) or RNeasy Plus Micro Kit (Qiagen, 74,034), respectively, according to the manufacturer’s protocol.

### Immunoblotting

For Western blotting, cells and neurons were lysed in Pierce IP lysis buffer (Thermo Scientific) supplemented with a phosphatase and protease inhibitor cocktail (Roche PhosSTOP and cOmplete, Mini, EDTA-free protease inhibitor cocktail). Cleared lysates were combined with sample buffer containing DTT and run on gradient proteins gels (Mini-PROTEAN TGX Precast Gels, Biorad) and transferred using the iBlot2 system. Following blocking, primary antibodies were applied- anti-STMN2 (rabbit, Proteintech, 10,586–1-AP), anti-SRSF7 (Rabbit, Proteintech, 11,044–1-AP), anti-GAPDH (clone 6C5 mouse, Millipore Sigma, MILL-CB1001), anti-neuron-specific beta-III Tubulin (Tuj-1; mouse, R&D systems, MAB1195), anti-TDP-43 C-terminal (rabbit, Proteintech, 12,892–1-AP)- then anti-mouse or anti-rabbit LICOR IRdye secondary antibodies. Signal detection and quantification was performed on the LICOR ODYSSEY system.

### Quantitative PCR

Reverse transcription of RNA to complementary DNA was performed using the iScript™ Advanced cDNA Synthesis Kit (Bio-Rad, 1,725,038). Quantitative RT-PCR (qRT-PCR) was performed with diluted cDNA, primers (synthesized by Integrated DNA Technologies), and the Maxima SYBR green qPCR master mix 2X (Thermo Fisher, K0253) with reactions run on a Real-Time PCR System (CFX96, Bio-Rad). All reactions were run in technical triplicates on the thermocycler and then averaged. Levels of all genes assayed were normalized to GAPDH expression unless noted otherwise. Normalized gene expression is presented as relative to the control sample. Primer pair sequences were obtained from prior publications or the MGH PrimerBank, and sequences are provided in the supplementary table.

### Lentiviral poly-PR and synthetic poly-PR

Lentiviral constructs to express STMN2-GFP, GFP, GFP-PR50 were described in Linares et al [33]. SRSF7-GFP (pLenti) to produce lentivirus was obtained from OriGene. The expected nuclear localization of this epitope-tagged SRSF7 was confirmed via fluorescent microscopy. For the transduction of neurons, high-titer lentiviral preparations from 293 T cells were performed by Alstem. Due to the higher expression of GFP alone, GFP-PR50 virus was used at twice the volume of GFP. PR20 was synthesized by Peptide 2.0 with purity > 95%, confirmed by HPLC, and dissolved in DMSO. Vehicle (DMSO) or PR20 peptide at a non-toxic concentration was added directly to neuron cell culture media.

### Axotomy assay

NGN2 hiPSC-derived neurons at day 5 post-differentiation were cultured on microfluidic devices (XONA Microfluidics SND150), mounted on glass coverslips pre-coated with 0.1 mg/mL poly-L-ornithine in 50 mM Borate buffer and 5ug/mL laminin, at a density of 100,000 neurons per device. GFP-PR50 or GFP lentivirus transduction was carried out in suspension during cell replating. A complete media change was executed the following day. Axotomy was conducted three days after plating in the microfluidic chambers by repeated vacuum aspiration following perfusion of 1X PBS in the axonal chamber. After axotomy, SiR-tubulin dye (NC0958386, Cytoskeleton) was diluted in fresh neuronal media. This dye allows for the observation of neurite morphology without the need for cell fixation, thereby avoiding potential confounding effects on cell morphology. Subsequently, images were taken 24 h post-axotomy using a widefield microscope at 20X magnification. The quantification of regrowing neurites, based on sirTubulin staining, was conducted using NeuronJ on ImageJ.

## Results

### *C9ORF72* repeat expansion-encoded poly-PR affects *STMN2* expression and axon regeneration

**Fig. 1:**
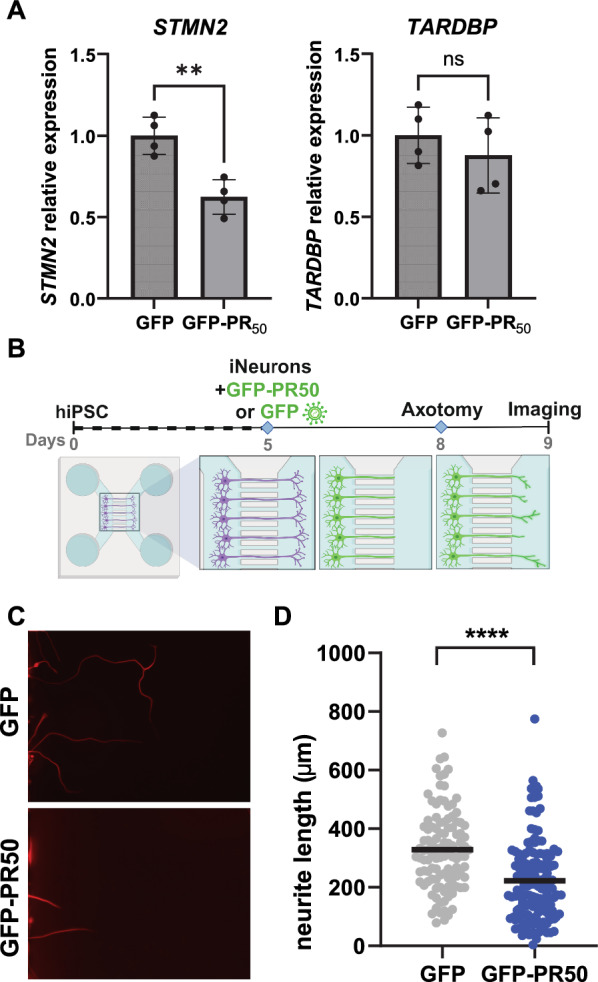
Poly-PR decreases *STMN2* expression in induced neurons and impairs axon regrowth. **A** Assessment of *STMN2* and *TARDBP* relative expression via qPCR in human induced neurons transduced with lentivirus expressing GFP-(PR)_50_ or GFP only and then collected 6 days later. (** = *p* < 0.01, two-tailed t-test; n = 4 from two independent differentiations). **B** Schematic of axotomy assay in induced neurons in microfluidics chambers. **C** Representative fluorescent images of neurite regrowth of induced neurons transduced with lentivirus for GFP-(PR)_50_ or GFP only. **D** Quantification of neurite regrowth from three devices for each condition. (GFP-(PR)_50_ n = 147, GFP *n* = 108; **** *p* < 0.0001, two-tailed *t*-test)

First, we asked whether a *C9ORF72* repeat expansion antisense-encoded dipeptide protein, poly-proline-arginine (poly-PR), could affect the expression of key ALS/FTD-associated transcripts. Human induced pluripotent stem cells (iPSCs) were differentiated into excitatory cortical-like patterned induced neurons through the expression of the neuralizing transcription factor NGN2 and patterning factors [32, 34, 35]. Induced neurons were transduced with lentiviruses to express poly-PR (GFP-PR_50_) or GFP alone. Poly-PR resulted in decreased abundance of the neural-enriched *STMN2* transcript compared to control, whereas *TARDBP* (encoding TDP-43) was unchanged (Fig. 1A). Additionally, induced neurons were treated with synthetic poly-PR_20_ peptide, which enters the nucleus [13, 36], or control (DMSO). PR_20_ also resulted in decreased *STMN2*, though did not reduce *UNC13A,* another disease-associated transcript [37] (Sup. Figure 1a). Of note, induction of the alternative *STMN2* cryptic exon (exon 2a)-containing transcript, which serves as a surrogate for reduced TDP-43 function [23, 24], was not detected following poly-PR treatment (Sup. Figure 1a). Moreover, in mining C9ORF72-related transcriptomic data by Kramer et al.[36], we noticed that poly-PR_20_ also appeared to result in reduced expression of *Stmn2* in murine primary cortical neurons (Sup. Figure 1b, c). As the TDP-43 cryptic exon-binding site in *STMN2* is not conserved in mice [26], this suggests that the effect of poly-PR on *STMN2* may be mediated in a distinct manner from the role of TDP-43 in the regulation of *STMN2* RNA processing.

*STMN2* encodes stathmin-2, also known as superior cervical ganglion-10, which is a neural-enriched microtubule binding protein that acts to maintain axons and promote axon regeneration following injury [21, 22]. A reduction of *STMN2* is sufficient to impair neurite regrowth after axotomy in human pluripotent stem cell-derived motor neurons [23, 24]. Given that poly-PR decreases *STMN2*, we sought to examine the functional consequence of this effect and hypothesized that this DPR may impair neural regeneration in vitro. Human induced neurons were plated in microfluidics devices that separate somas and axons, which grow and project to the distal compartment through microchannels, and then transduced with lentivirus expressing GFP-PR_50_ or GFP alone (Fig. 1b). Axons were removed from the distal compartment and labelled with a soluble fluorescent dye to monitor regrowth, avoiding immunostaining and processing that could disrupt neurite morphology. We observed that poly-PR markedly impaired axon regrowth relative to control following axotomy [GFP: 328.7 ± 134.5 um, PR50: 221.9 ± 135.0 um (mean ± SD); p-val. < 0.001] (Fig. 1c-d). This result suggests that poly-PR reduces the regenerative capacity of excitatory neurons and provides an additional mechanism by which the pathogenic C9ORF72 repeat expansion may gradually impair neural function, especially following nerve injury.

### Phospho-proteomics reveals that poly-PR perturbs nuclear RNA-binding proteins

**Fig. 2:**
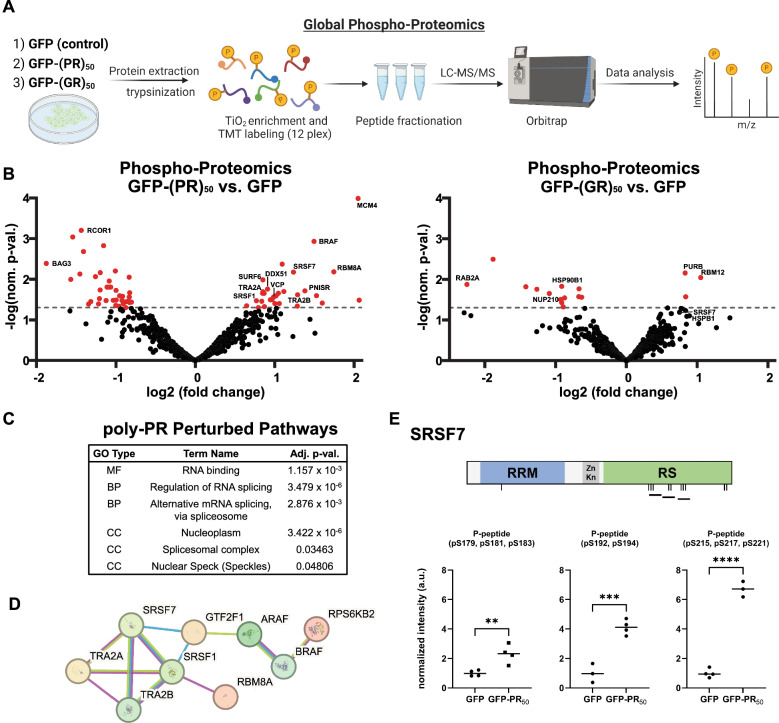
Global phospho-proteomics reveals that poly-PR perturbs phospho-proteins involved in RNA processing. **A** Global phospho-proteomics mass spectrometry workflow of cells expressing GFP (control), GFP-(PR)_50_, GFP-(GR)_50_, collected from 4 independent transfections. **B** Volcano plot of the relative abundance of phospho-proteins from cells expressing GFP-(PR)_50_ versus GFP (control) and GFP-(GR)_50_ vs. GFP (control). **C** Gene ontology (GO) enrichment analysis of phospho-proteins increased in abundance in GFP-(PR)_50_, categorized by molecular function (MF), biological process (BP), or cellular component (CC). **D** Protein–protein interaction network of phospho-proteins increased in GFP-(PR)_50._ **E** Schematic of phosphorylation sites for the RNA processing factor SRSF7; RRM: RNA recognition motif, Zn Kn: Zinc knuckle domain, RS: Arginine/serine-rich domain. Changes in the relative abundance of selected SRSF7 phospho-peptides in GFP-(PR)_50_ (*n* = 3–4 per condition)

To further understand how poly-PR affects cellular processes, such as mRNA processing, that might mediate *STMN2* expression and axon repair, we undertook an unbiased global phospho-proteomics approach. We reasoned that DPRs could perturb disease-related pathways in ways that may not be completely reflected in transcriptomics or by the assessment of protein abundance alone [38, 39]. Cells were transfected to express GFP-PR_50_, GFP-GR_50_ for comparison, or GFP alone in four independent experiments. Following protein processing, phospho-peptides were enriched using titanium dioxide (TiO2) affinity chromatography with tandem mass tag (TMT) labeling of phospho-peptides for quantitative mass spectrometry (LC–MS/MS) (Fig. 2a). This approach led to the detection of over 4,500 phospho-peptides from 1,565 phospho-proteins and, following stringent analysis, resulted in the relative quantification of 597 phospho-proteins across all conditions.

Among the 27 phospho-proteins that were relatively increased in abundance in the presence of poly-PR, gene ontology (GO) analysis demonstrated clear enrichment for RNA binding proteins that function in mRNA processing and particularly those associated with the cellular compartments of the nucleolus and nuclear speckles (Fig. 2b-d). The preferential effect of poly-PR on nuclear-localized proteins is consistent with its well-characterized subcellular distribution in several model systems [6, 13, 40]. For instance, poly-PR localizes to the granular component of the nucleolus [14], where it disrupts an interaction between nucleophosmin (NPM1) and the C-terminal arginine-rich region of SURF6 [41]. Consistent with this finding, we observed that poly-PR, though not poly-GR, induced significant dysregulation of SURF6 phosphorylation. Among the most perturbed phospho-proteins in the presence of poly-PR were two members of the SRSF (serine/arginine-rich splicing factors) family, SRSF7 and SRSF3. SRSFs play several roles in mRNA processing, including constitutive and alternative splicing and alternative polyadenylation [42]. It has been well-described that the roles of SRSF proteins in mRNA processing are dynamically regulated through phosphorylation of their low-complexity C-terminal arginine/serine-rich (RS) domains, which acts to inhibit their activity [43]. For SRSF7, poly-PR led to differential changes in the abundance of phospho-peptides residing specifically within its RS domain as opposed to its N-terminal RNA recognition motif (RRM) or zinc-knuckle domain (Fig. 2e). Of note, genome-wide CRISPR-based screens in human embryonic stem cells found that several SRSF genes, including *SRSF7*, were among core essential fitness genes for stem cells [44], consistent with *Srsf7* being essential for viability in mice [45]. In contrast to poly-PR, poly-GR appeared to have modest effects on the phospho-proteome in this context. Phospho-proteins that were increased in abundance included RBM12, an RNA-binding protein associated with psychiatric disease, and those decreased included HSP90B1, a member of the HSP90 family protein chaperone, which can modify poly-GR toxicity [46]; though, enrichment analysis did not pinpoint any particular biological processes under this condition (Fig. 2B). Our findings suggest that poly-PR perturbs the function of selected RNA processing factors.

To confirm that increased phosphorylation of RBPs was not due to changes in their overall abundance, we also performed TMT-based proteomics to globally assess protein levels (without enrichment for phospho-peptides). This approach provided quantification of over 4,300 proteins in cells expressing GFP-PR or GFP only control (Sup. Figure 2A). In the presence of poly-PR, upregulated proteins were significantly enriched for mitochondrial proteins (GO:0005739: mitochondrion, adj. p val.: 2.152 × 10^–8^), suggesting a relative increase in mitochondria, though not for RNA processing related terms. Interestingly, the protein that was most significantly increased was superoxide dismutase 1 (SOD1) (Sup. Figure 2B), and prior studies have suggested partial overlap between the cellular pathways altered in models of SOD1-ALS and C9ORF72-ALS [47]. Notably, there were no significant changes in the abundance of TDP-43 and SRSF7 (Sup. Figure 2B).

To test whether changes in RBP phosphorylation was associated with altered gene expression, we performed quantitative PCR to assess levels of their corresponding transcripts in the presence of DPRs. In general, poly-PR did not result in significant changes in these transcripts, suggesting that its effect on the corresponding RNA binding proteins is post-transcriptional (Sup. Figure 3a). Interestingly, both poly-GR and poly-PR resulted in significantly increased expression of *SRSF1*, encoding an essential pre-mRNA splicing factor [42]. Also, poly-GR, though not poly-PR, lead to increased expression of *SRSF7*, suggesting that poly-GR may evoke a transcriptional response related to RNA processing (Sup. Figure 3a-c). However, protein levels of endogenous SRSF7 were not increased upon expression of poly-GR, poly-PR, or poly-GA (Sup. Figure 3a-b). Of note, there was no apparent gel mobility-shift in SRSF7 with DPR expression, although this is not surprising as SRSF proteins are well-established to have high levels of constitutive phosphorylation [42, 48]. Overall, phospho-proteomics revealed poly-PR-dependent perturbations in nuclear-localized proteins that were not detectable in prior transcriptomic studies, particularly the dysregulation of several RNA processing factors.

**Fig. 3:**
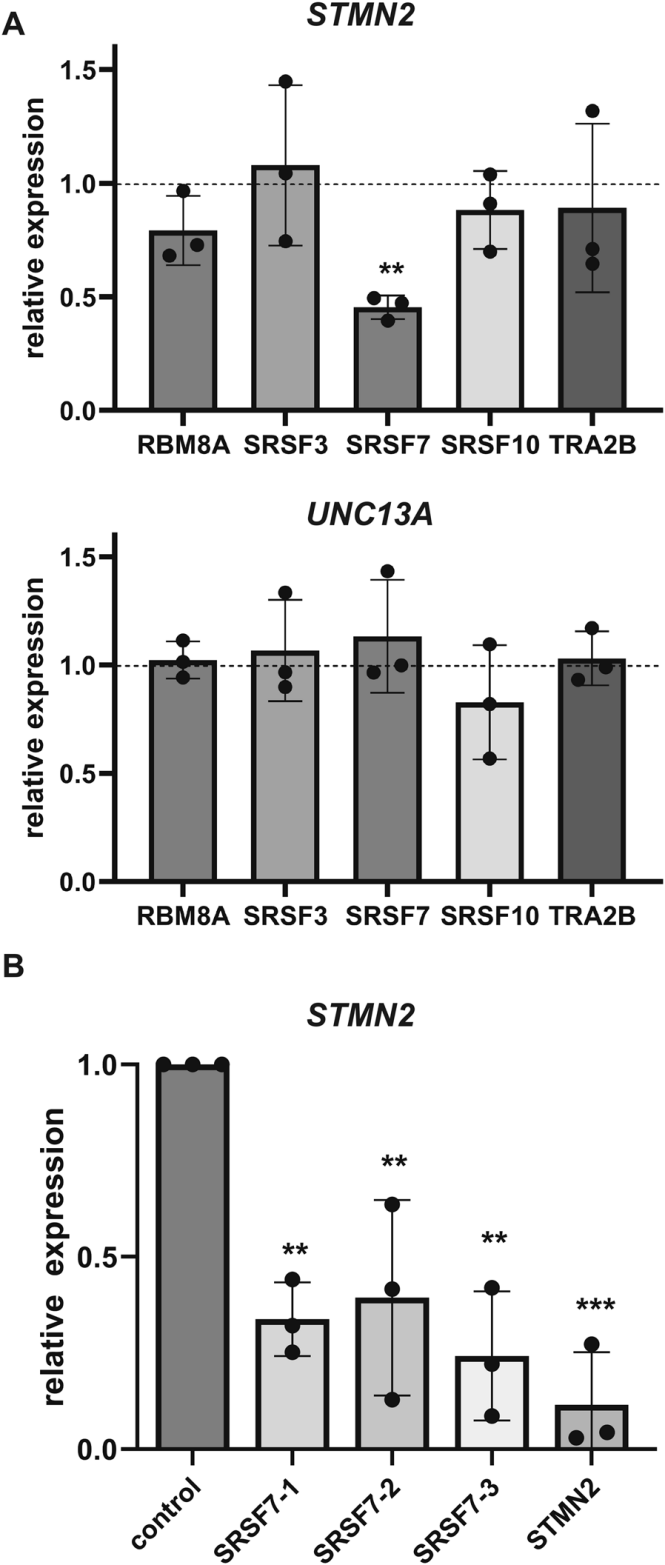
Knockdown of essential RNA processing factor SRSF7 decreases expression of STMN2 in induced neurons. **A** Representative RNA binding proteins identified from the global phospho-proteomics analysis were depleted in induced neurons, and expression of *STMN2* and *UNC13A* were assessed via qPCR compared to non-targeting control (*n* = 3, ** = *p* < 0.01, unpaired two-tailed *t*-test). **B** *STMN2* expression following transfection of induced neurons with non-targeting control siRNA or three individual siRNAs targeting *SRSF7* across three independent differentiations. Pooled siRNAs against *STMN2* served as a positive control. (** = *p* < 0.01, *** = *p* < 0.001, one-way ANOVA with Tukey’s correction for multiple comparisons)

### SRSF7 sustains levels of STMN2 in human stem cell-derived induced neurons

We next sought to determine whether these RNA binding proteins, especially members of the SRSF protein family, could affect the transcript levels of key neural-enriched ALS/FTD-associated transcripts [21]. We tested the effects of siRNA-mediated knockdown of several essential RNA processing factors on the expression of *STMN2* and *UNC13A* transcripts in induced neurons across independent differentiations. Reduced expression of SRSF7 consistently led to decreased expression of *STMN2* but not *UNC13A* (Fig. 3a). In contrast, depletion of other selected RNA binding proteins, including SRSF10 and TRA2B, did not have a significant effect on *STMN2* or *UNC13A*. Considering the conserve effect, we also assayed the effect of increasing SRSF7. Overexpression of SRSF7 did not significantly affect *STMN2* expression, which is not entirely unexpected as this SRSF protein is one component of complex RNA processing machinery in the nucleus (Sup. Figure 1D). Overall, this suggests that SRSF7 is a novel regulator of *STMN2* in human neurons.

To validate this finding, induced neurons were derived from a different genetic background, the well-characterized KOLF2.1 J human iPS cell line [49]. All three individual siRNAs targeting *SRSF7* consistently reduced the expression *STMN2* in induced neurons, and siRNAs targeting *STMN2* was included as a positive control (Fig. 3b). Given the defined relationship between TDP-43 and *STMN2* [25], one possibility was that reduced SRSF7 could somehow perturb TDP-43 function and hence induce the expression of the STMN2 cryptic exon. Therefore, we also assessed the *STMN2* CE (exon 2A) but did not detect induction of this transcript in the presence of SRSF7 knockdown (not shown). Of note, we confirmed this assay whereby knockdown of *TARDBP* robustly increased *STMN2 CE* in induced neurons (Sup. Figure 1c), consistent with the original findings in hPSC-derived lower motor neurons [23].

### SRSF7 supports axonal regeneration in induced neurons

**Fig. 4:**
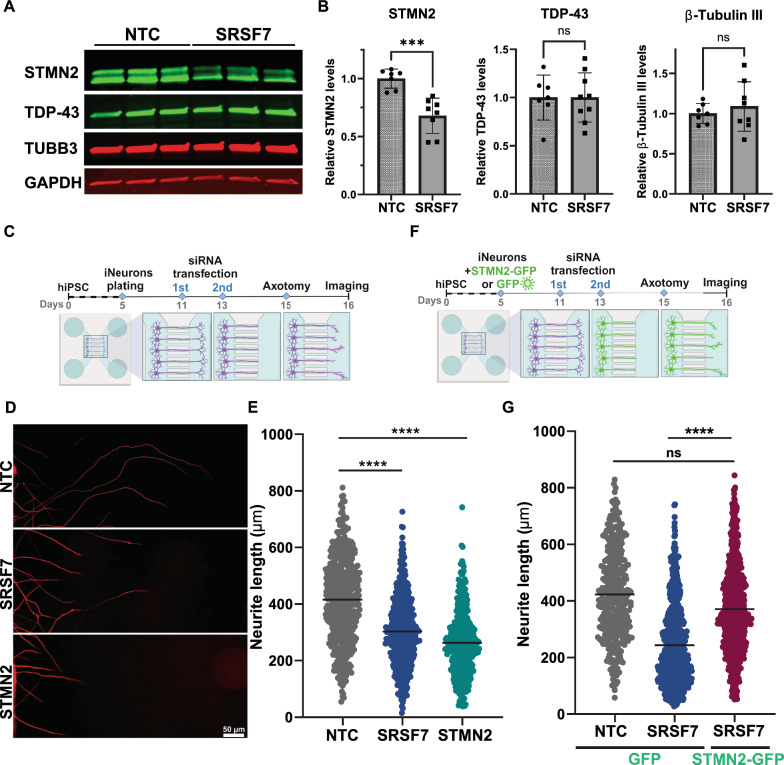
Knockdown of essential SRSF7 reduces levels of STMN2, though not TDP-43, and impairs axon regrowth in induced neurons. **A** Representative immunoblot of STMN2, TDP-43, beta-tubulin IIIb (Tuj1) from induced neurons following depletion of SRSF7 or a non-targeting control. **B** Quantification of the levels of STMN2, TDP-43, and Tuj1 normalized to GAPDH, from three independent experiments (*n* = 7–8, *** = *p* < 0.001, two-tailed *t*-test). **C** Experimental workflow for axotomy assay. **D** Representative images of neurite regrowth following transfection of siRNAs targeting *STMN2*, *SRSF7*, or non-targeting control. **E** Axotomy assay with quantification of neurite regrowth from three separate devices for each condition (one-way ANOVA, **** = *p* < 0.0001). **F** Experimental workflow for rescue axotomy assay. **G** Axotomy assay of neurons transduced with lentivirus expressing STMN2-GFP compared to GFP control following siRNA transfection of non-targeting control or SRSF7 siRNA. Quantification of neurite regrowth, results from multiple separate devices for each condition (one-way ANOVA, **** = *p* < 0.0001)

To further characterize this finding, we asked whether SRSF7 regulates the levels of STMN2 protein. We found that depletion of SRSF7 lead to decreased STMN2 and appeared to affect both major isoforms of this protein and, importantly, without changes in the abundance of TDP-43 in neurons (Fig. 4a-b). Given that STMN2 is a microtubule binding, we also examined the expression of beta-tubulin III, an abundant neural-enriched component of microtubules, and observed no significant changes following SRSF7 depletion (Fig. 4a-b). This is consistent with a recent report of a *Stmn2* knockout mouse model, where loss of Stmn2 did not affect the overall level of β-III tubulin in the spinal cord, and total β-III tubulin levels were constant between wild-type and STMN2-null hPSC-derived motor neurons [26].

Since SRSF7 maintains STMN2 levels in neurons, we wondered whether this RNA binding proteif n similarly plays a supportive role in axonal regeneration. Human induced neurons were plated in microfluidic devices for transfection followed by an axotomy assay (Fig. 4c,d). Following quantification, we consistently observed that knockdown of *SRSF7* reduced axon regrowth relative to the nontargeting control (p < 0.001), and to a similar extent as targeting *STMN2* itself as a positive control (mean non-targeting control: 415.7 µm, *SRSF7:* 303.3 µm, *STMN2*: 263.3 µM; Fig. 4e). To determine whether this effect of SRSF7 loss of function could be attributed to diminished STMN2, we performed a rescue experiment whereby supplemental expression of exogenous STMN2-GFP or GFP alone were provided to neurons prior to performing axotomy (Fig. 4f). In this approach, we observed that the presence of increased STMN2 significantly mitigated the effects of SRSF7 loss of function on axon regrowth (p < 0.001) (Fig. 4g). This suggests that SRSF7 regulates the regenerative capacity of human neurons, at least in part, due to its role in sustaining the expression of *STMN2*.

## Discussion

Although C9ORF72 DPRs consistently demonstrate detrimental effects across several model systems [10], two early-stage randomized clinical trials (NCT03626012, NCT04931862) in C9-ALS patients have recently determined that antisense oligonucleotides (ASOs) targeting the sense-encoded repeat-containing transcript were not beneficial, and might even have adverse consequences [50]. While there are many possible explanations for these findings, they have cast some doubt as to the contributions of sense-encoded DPRs, such as poly-GR, as drivers of disease progression [51]. One possibility is that anti-sense encoded DPRs, particularly poly-PR, may have greater toxic effects than sense-encoded DPRs [40]. Moreover, reducing expression of the sense-encoded repeat transcript may inadvertently result in the upregulation of anti-sense repeat-containing transcripts, and hence could act to increase the abundance of poly-PR, which currently eludes validated assays for the detection of DPRs in biofluids [52].

Here, we uncovered a novel link between poly-PR and the regulation of *STMN2*, providing a potential gain-of-function mechanism by which an anti-sense-encoded DPR may contribute to disease pathogenesis. Applying an unbiased global phospho-proteomics approach, we found that poly-PR perturbs the phosphorylation status of RNA processing factors, including serine/arginine-rich splicing factor 7 (SRSF7). Furthermore, we demonstrate that loss of SRSF7 function results in reduced levels of STMN2, a critical regulator of microtubules and axon homeostasis [21, 27, 28], and a corresponding impairment of neural recovery upon injury.

Both poly-GR and poly-PR have been implicated in the disruption of membraneless organelles, including nuclear speckles, which contain SRSF RNA binding proteins [13, 14]. One study that used proteomics (IP-MS) to identify candidate DPR interacting proteins suggested that poly-PR interacts with SRSF7 [14], though another similarly designed study did not detect this interaction [53]. Our unbiased phospho-proteomics indicates that poly-PR, in contrast to poly-GR, perturbs the functional status of SRSF7 and additional RBPs, which could be related to the impairment of transport to or from nuclear speckles. Our results provide a further connection between poly-PR gain-of-function effects and dysregulation of gene expression, especially in the cellular context of cortical-like neurons.

Reducing TDP-43 in human stem cell-derived motor neurons and in neuroblastoma cells results in reduced expression of STMN2 and the induction of an aberrant truncated *STMN2* transcript [23, 24]. The expression of the *STMN2* cryptic exon clearly correlates with TDP-43 pathology in several neurodegenerative diseases [25, 30]. However, at least in FTD patients, the burden of TDP-43 pathology in the frontal cortex does not correlate with the expression levels of full-length *STMN2* [30], raising the possibility that additional pathways contribute to dysregulation of *STMN2* expression. We propose that SRSF7 is a novel regulator of *STMN2* in neurons, acting independently of TDP-43, and loss of SRSF7 function could contribute to disease.

What concordant evidence for dysregulation of SRSF7 can be found in ALS/FTD patients? Preliminary results of comprehensive cellular level multi-omics analysis of C9-ALS and sporadic ALS brains from McKeever et al. highlight potential disease relevance for SRSF7 [54]. In an unbiased manner, the authors applied a deep learning model to identify RNA binding proteins (RBPs) that may be associated with transcriptional dysregulation in neurons from post-mortem cortical samples. Among 89 RBP implicated in disease, SRSF7 appeared to be the most significantly upregulated one in both C9-ALS and in sporadic ALS- specifically in the cellular context of excitatory neurons [54]. Given that SRSF7 maintains its expression through a negative feedback mechanism [55], it is plausible that this increase in SRSF7 expression represents a compensatory response to loss of SRSF7 function in C9-ALS. Taken together with previous studies, our findings suggest that both TDP-43 and SRSF7 might functionally converge upon the neural-enriched *STMN2* transcript in disease. Of note, SRSF7 has been previously implicated in the alternative splicing of *MAPT* exon 10, which is dysregulated in tau-associated forms of FTD [56].

SRSF proteins play multiple roles in RNA processing, including alternative splicing and alternative polyadenylation, and are regulated by complex levels of phosphorylation by many protein kinases. The CLKs (Cdc2-like kinases, CLK1-4) phosphorylate SRSFs in the nucleus, especially serine residues within RS domains in a processive manner, targeting multiple sites without immediate dissociation [57]. Although CLKs are ubiquitously expressed, selective CLK kinase inhibitors are being explored in clinical trials for certain types of cancer [58]. Our results suggest that further studies may be needed to examine the function of CLKs in neurons and to examine the feasibility of CLK inhibitors as a means to modulate gene expression, including *STMN2*, to combat neurodegenerative processes. In transcriptome-wide RNA-binding profiles of selected SRSF proteins conducted in a murine embryonic carcinoma cell line, we noticed that SRSF7 interacts with *Stmn2* based on iCLIP (individual-nucleotide resolution UV crosslinking and immunoprecipitation) results [59]. We expect “hyper-phosphorylation” of SRSF7 would be associated with impairment of the activity of SRSF7 in RNA processing, equivalent to a reduction of SRSF7 levels, based on studies of SRSFs in other model systems [60–62]. However, one limitation of our study is that changes in the phosphorylation of residues within its RS domain could be associated with other molecular phenotypes. Further studies will be necessary to define the downstream consequences at specific phosphorylation sites in SRSF7 and related proteins.

Additional members of the SRSF family of RBPs may modify other facets of C9ORF72-associated pathogenesis. SRSF1 was identified as a suppressor of neurodegeneration in C9ORF72-related *Drosophila* models of disease, and this factor regulates the trafficking of C9ORF72 repeat transcripts [63, 64]. We observed that both poly-GR and poly-PR lead to increase expression of *SRSF1*, which could promote the production of DPRs. Additional studies will be necessary to dissect the interaction between SRSF7 and STMN2 and the role of SRSFs in RNA processing in human neurons.

## Conclusions

Our study provides a novel phospho-proteomic dataset that reveals how C9-ALS/FTD-associated poly-PR perturbs the phosphorylation status of several RBPs linked to nuclear speckles and mRNA processing. Although there remain limitations related to studying exogenous DPRs in model systems, we observed that both poly-PR and the RNA processing factor SRSF7 affect the expression of neural-enriched STMN2 in human stem cell-derived excitatory neurons. Given that TDP-43 acts to maintain STMN2 levels through the suppression of an aberrant cryptic splicing event, our findings suggest that there are additional disease-relevant mediators of *STMN2* expression. Consequently, it will be important to further study the regulation of *STMN2*, which has emerged as a potential therapeutic target for ALS.

## Supplementary Information

Supplementary file 1.

Supplementary file 2.

Supplementary file 3.

Supplementary file 4: Figure S1: C9ORF72-associated poly-PR reduces STMN2 expression in neurons. A) Assessment of STMN2 and UNC13A via qPCR in induced neurons following treatment with poly-(PR)¬20 peptide or vehicle (DMSO) for 36 hours (* = p <0.05, two-tailed t-test). B) Prior bulk RNA-sequencing results by Haney et al. captured changes in Stmn2 and Unc13a expression in primary cortical neurons treated with poly-(PR)¬20 peptide compared to untreated neurons. C) Representative qPCR for detection of the STMN2 transcript containing alternative “cryptic” exon 2a from induced neurons following siRNA knockdown of selected RNA binding proteins and non-targeting control (NTC). D) Induced neurons were transduced with lentivirus to express GFP or SRSF7-GFP and then harvested 7 days later, followed by qPCR for STMN2.

Supplementary file 5: Figure S2: Global proteomics for poly-PR. A) Volcano plot of the relative abundance of proteins from cells expressing GFP-(PR)50 vs. GFP (control) from LC-MS/MS proteomics (n=3 biological replicates). Note, the abundance of detected GFP was approximately twice for GFP alone compared to GFP-PR in the proteomic results. B) Normalized protein abundance of selected proteins, including disease-associated SOD1 and TDP-43 (unpaired t-test, *** p < 0.001).

Supplementary file 6: Figure S3: Effect of DPRs on SRSF7 protein level and selected transcripts encoding RNA binding proteins. A) Immunoblot of SRSF7 protein from cells transfected with DPRs: GFP-(GA)50, GFP-(GR)50, GFP-(PR)50 or GFP alone (control). B) Quantification of SRSF7 levels normalized to GAPDH from three independent transfections (two-tailed t-test, ns: not significant). C) Quantitative PCR for selected transcripts encoding RNA binding proteins normalized to housekeeping genes (GAPDH, ACTB) in cells transfected with DPRs or GFP alone (control) from three independent transfections. (Two-way ANOVA with Dunnett’s multiple comparisons test; * adj. p < 0.05, ** adj. p < 0.01, *** adj. p < 0.001, else non-significant).

## Data Availability

Data is provided within the manuscript or supplementary information files.

## Abbreviations

ALS: Amyotrophic lateral sclerosis
ASO: Antisense oligonucleotide
C9ORF72: Chromosome 9 open reading frame 72
CE: Cryptic exon
DPR: Dipeptide repeat protein
FTD: Frontotemporal dementia
iPSC: Induced pluripotent stem cell
NGN2: Neurogenin 2
Poly-GR: Poly-glycine-arginine protein
Poly-PR: Poly-proline-arginine protein
qPCR: Real-time quantitative polymerase chain reaction
RBP: RNA binding protein
RS: Arginine/serine-rich
SRSF7: Serine/arginine-rich splicing factor 7
TDP-43: TAR DNA-binding protein 43
