# Supplementary material for: C9ORF72 poly-PR disrupts expression of ALS/FTD-implicated STMN2 through SRSF7
Source: Acta Neuropathol Commun. 2025 Mar 26;13:67. doi: 10.1186/s40478-025-01977-2 (PMC11948778; doi:10.1186/s40478-025-01977-2)
Supplement: Supplementary file 2 [file 40478_2025_1977_MOESM2_ESM.pdf]

Supplemental Table 1: Quantitative PCR Primers

STMN2
STMN2_ex1F: GACTCAGTGCCTTATTCAGT
STMN2_ex2R: GTTGATGTTGCGAGGTTCCG
(reference: Melamed et al., 2019)
STMN2 CE
STMN2_ex1F: GACTCAGTGCCTTATTCAGT
STMN2_ex2aR: TTTCTCTCGAAGGTCTTCTG
(reference: Melamed et al., 2019)
UNC13A
UNC13A_F: GGACGTGTGGTACAACCTGG
UNC13A_R: GTGTACTGGACATGGTACGGG
(reference: Ma et al., 2022)
RBM8A
RBM8Af: GATGGGGACGAGAGCATTCAC
RBM8Ar: CGCTGTCATAATCCTCACGCA
SF3B4
SF3B4f: GCATCAGCTCACAACAAAAACC
SF3B4r: GCAACTTCTCATCAATCTCAGGG
SF3B5
SF3B5f: ACTGACCGCTACACCATCCAT
SF3B5r: GTAGTTGAGAAGGTCGAAGTGG
SRSF1:
SRSF1f: GCCGCATCTACGTGGGTAAC
SRSF1r: GAGGTCGATGTCGCGGATAG
SRSF3
SRSF3f: ATGGAAGAACACTATGTGGCTG
SRSF3r: GGGACGGCTTGTGATTTCTCT
SRSF7
SFSR7f: GGAAAGGTGATTTGTGGCTCC
SFSR7r: GGTCTATCAAAACGTGATCTCCG
SRSF10
SRSF10f TGAGGATGTTCGTGATGCTGA
SRSF10r CCTCCTTTCATAACTTCGGCTT
GAPDH
GAPDHf: GTTCGACAGTCAGCCGCATC
GAPDHr: GGAATTTGCCATGGGTGGA
ACTB
ACTBf: AGATCAAGATCATTGCTCCTCCT
ACTBr: CGGACTCGTCATACTCCTGC
