# Supplementary figures and images for: C9ORF72 poly-PR disrupts expression of ALS/FTD-implicated STMN2 through SRSF7
Source: Acta Neuropathol Commun. 2025 Mar 26;13:67. doi: 10.1186/s40478-025-01977-2 (PMC11948778; doi:10.1186/s40478-025-01977-2)

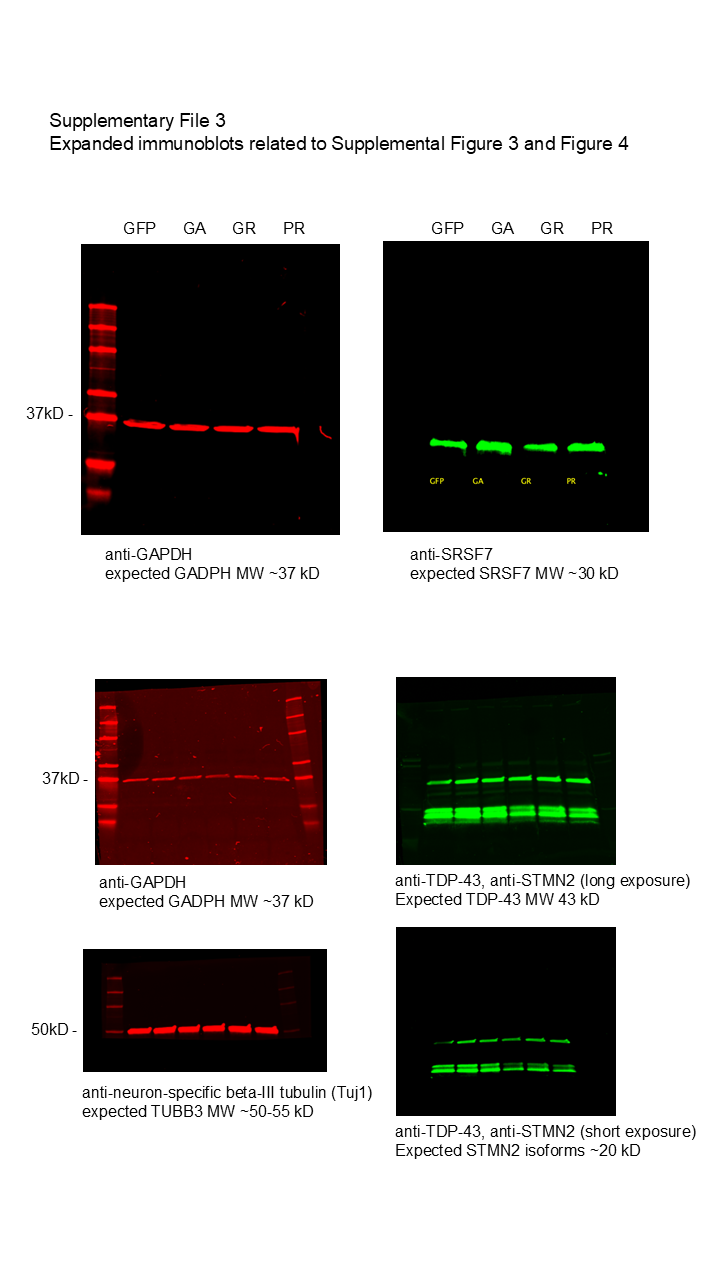

Supplement: Supplementary file 3 [file 40478_2025_1977_MOESM3_ESM.tif]

A

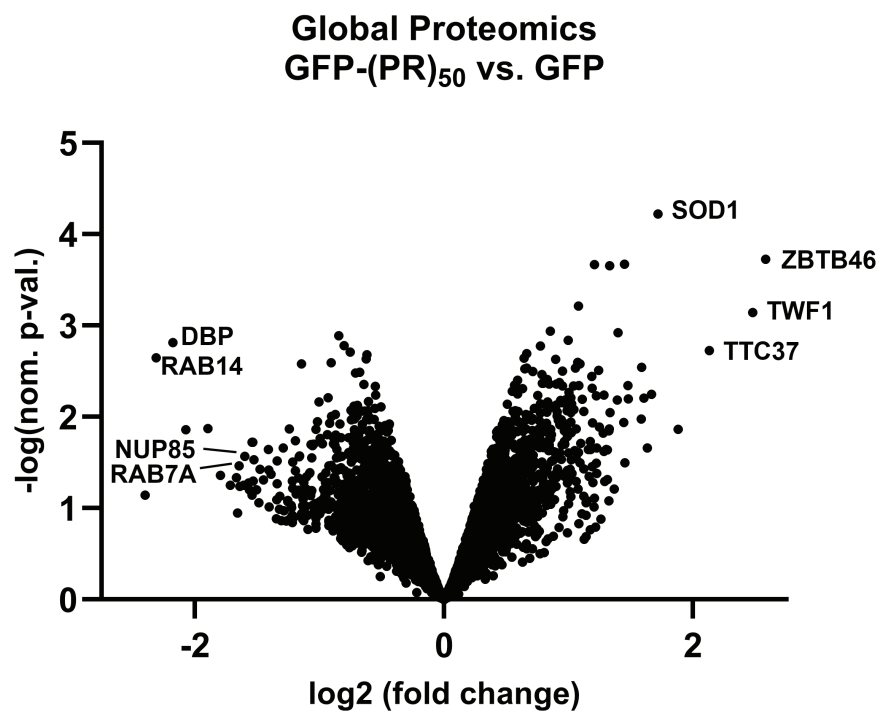

B

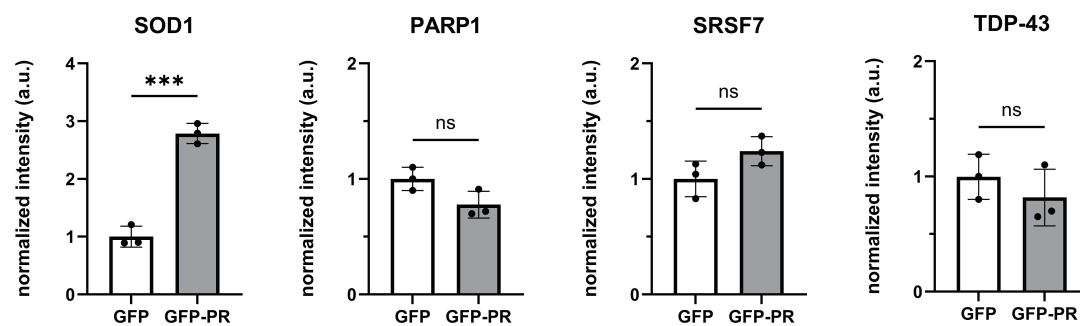

Supplement: Supplementary file 5 — Figure S2: Global proteomics for poly-PR. A) Volcano plot of the relative abundance of proteins from cells expressing GFP-(PR)50 vs. GFP (control) from LC-MS/MS proteomics (n=3 biological replicates). Note, the abundance of detected GFP was approximately twice for GFP alone compared to GFP-PR in the proteomic results. B) Normalized protein abundance of selected proteins, including disease-associated SOD1 and TDP-43 (unpaired t-test, *** p < 0.001). [file 40478_2025_1977_MOESM5_ESM.pdf]

A

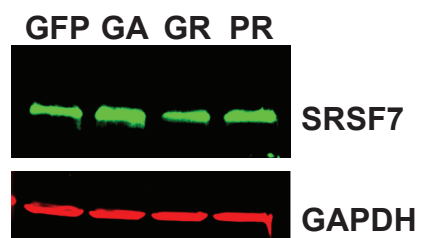

B

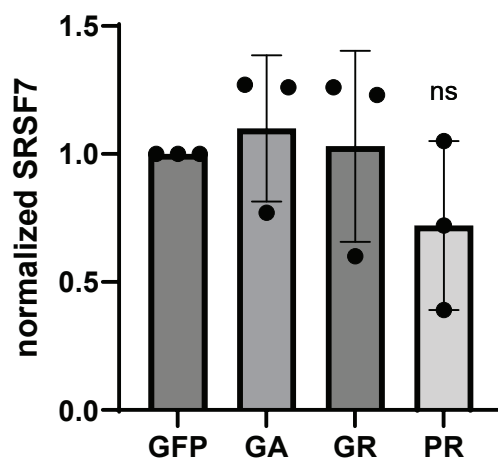

C

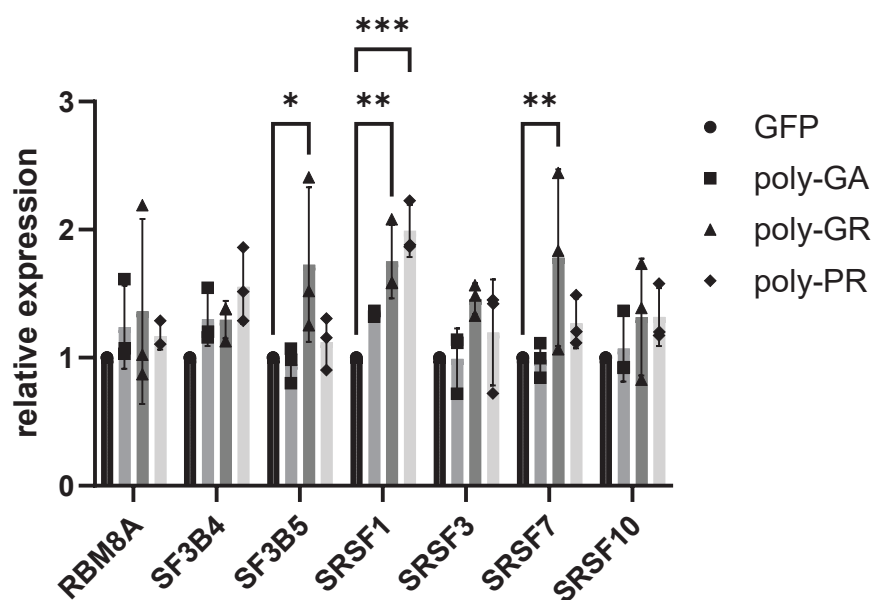

Supplement: Supplementary file 6 — Figure S3: Effect of DPRs on SRSF7 protein level and selected transcripts encoding RNA binding proteins. A) Immunoblot of SRSF7 protein from cells transfected with DPRs: GFP-(GA)50, GFP-(GR)50, GFP-(PR)50 or GFP alone (control). B) Quantification of SRSF7 levels normalized to GAPDH from three independent transfections (two-tailed t-test, ns: not significant). C) Quantitative PCR for selected transcripts encoding RNA binding proteins normalized to housekeeping genes (GAPDH, ACTB) in cells transfected with DPRs or GFP alone (control) from three independent transfections. (Two-way ANOVA with Dunnett’s multiple comparisons test; * adj. p < 0.05, ** adj. p < 0.01, *** adj. p < 0.001, else non-significant). [file 40478_2025_1977_MOESM6_ESM.pdf]
