# Supplementary material for: C9ORF72 poly-PR disrupts expression of ALS/FTD-implicated STMN2 through SRSF7
Source: Acta Neuropathol Commun. 2025 Mar 26;13:67. doi: 10.1186/s40478-025-01977-2 (PMC11948778; doi:10.1186/s40478-025-01977-2)

A

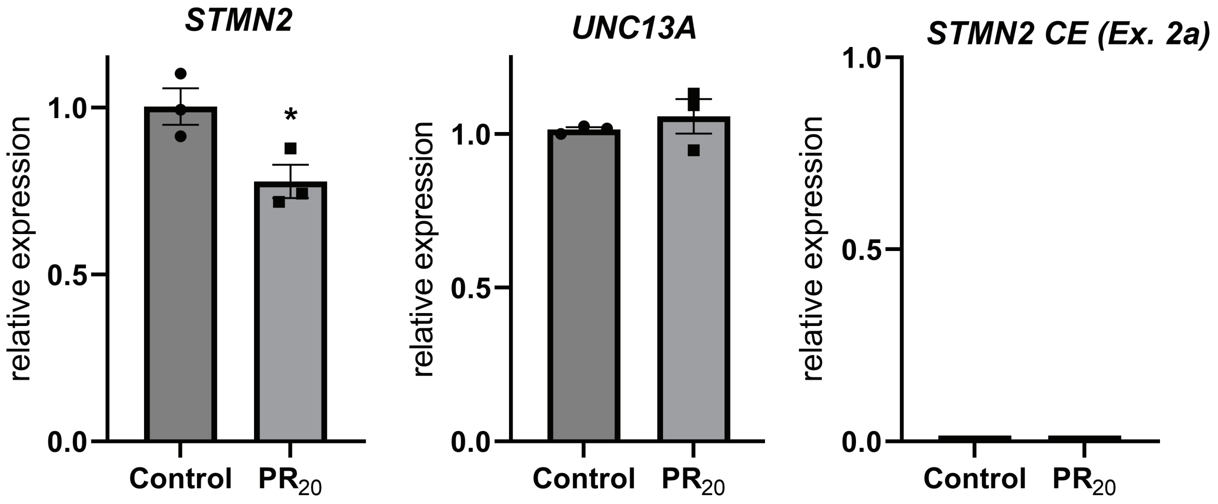

B

Haney et al.
RNA-sequencing of PR20 treated vs. untreated primary neurons:

Stmn2: -0.4894 (log2 fold change); p. adj.: 7.08E-11
Unc13a: 0.3993 (log2 fold change); p. adj.: 0.04134

C

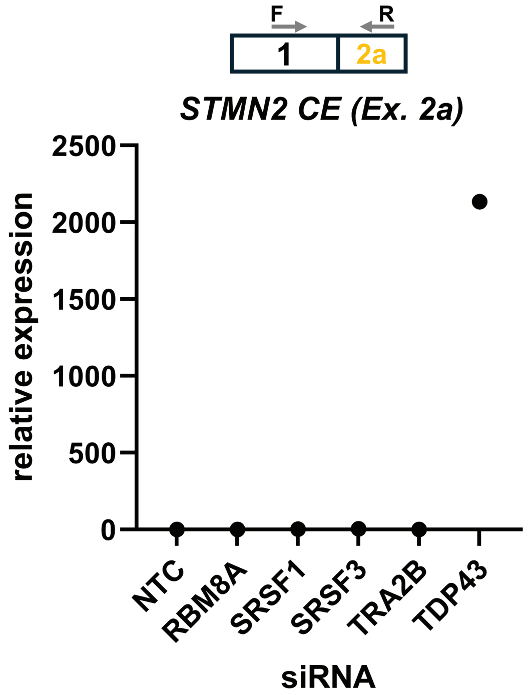

D

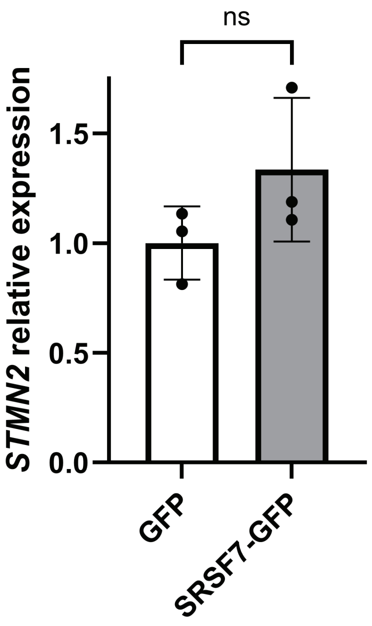

Supplement: Supplementary file 4 — Figure S1: C9ORF72-associated poly-PR reduces STMN2 expression in neurons. A) Assessment of STMN2 and UNC13A via qPCR in induced neurons following treatment with poly-(PR)¬20 peptide or vehicle (DMSO) for 36 hours (* = p <0.05, two-tailed t-test). B) Prior bulk RNA-sequencing results by Haney et al. captured changes in Stmn2 and Unc13a expression in primary cortical neurons treated with poly-(PR)¬20 peptide compared to untreated neurons. C) Representative qPCR for detection of the STMN2 transcript containing alternative “cryptic” exon 2a from induced neurons following siRNA knockdown of selected RNA binding proteins and non-targeting control (NTC). D) Induced neurons were transduced with lentivirus to express GFP or SRSF7-GFP and then harvested 7 days later, followed by qPCR for STMN2. [file 40478_2025_1977_MOESM4_ESM.pdf]
